# Factors affecting the psychosocial well-being of orphan and separated children in five low- and middle-income countries: Which is more important, quality of care or care setting?

**DOI:** 10.1371/journal.pone.0218100

**Published:** 2019-06-13

**Authors:** Hy V. Huynh, Susan P. Limber, Christine L. Gray, Martie P. Thompson, Augustine I. Wasonga, Vanroth Vann, Dafrosa Itemba, Misganaw Eticha, Ira Madan, Kathryn Whetten

**Affiliations:** 1 Center for Health Policy & Inequalities Research, Duke Global Health Institute, Duke University, Durham, North Carolina, United States of America; 2 Department of Youth, Family & Community Studies, Clemson University, Clemson, South Carolina, United States of America; 3 Ace Africa Kenya, Bungoma, Kenya; 4 Development for Cambodian Children (DCC), Battambang, Cambodia; 5 Tanzania Women Research Foundation (TAWREF), Moshi, Tanzania; 6 Stand for Vulnerable Organization (SVO), Addis Ababa, Ethiopia; 7 Sahara Centre for Residential Care & Rehabilitation, New Delhi, India; University of Botswana, BOTSWANA

## Abstract

As millions of children continue to live without parental care in under-resourced societies in low- and middle-income countries (LMICs), it is important for policymakers and practitioners to understand the specific characteristics within different care settings and the extent to which they are associated with outcomes of orphan and separated children (OSC). This study was designed to (1) examine if the psychosocial well-being of OSC in under-resourced societies in LMICs is more dependent on the availability of certain components of quality of care rather than the care setting itself (i.e. the residential care-based or community family-based setting), and (2) identify the relative significance of certain components of quality of care that are associated with a child’s psychosocial well-being across different OSC care settings. This study drew from 36-month follow-up data from the Positive Outcomes for Orphans (POFO) Study and used a sample population of 2,013 (923 institution- and 1,090 community-based) OSC among six diverse study sites across five LMICs: Cambodia, India (Hyderabad and Nagaland), Kenya, Tanzania, and Ethiopia. Analyses showed that all four components of quality of care significantly predicted child psychosocial well-being. Child psychosocial well-being across “high” and “low” levels of quality of care showed negligible differences between residential- and community-based care settings, suggesting the important factor in child well-being is quality of care rather than setting of care. Practical and policy implications and future research are discussed.

## Introduction

Global, national, and local leaders are struggling to find care solutions for the estimated 140,000,000 children worldwide who have lost one or both parents and millions more who have been separated from both parents (hereafter defined as orphan and separated children [OSC]).[1] High mortality among young adults from conditions such as malaria, tuberculosis, HIV/AIDS, pregnancy complications, accidents and natural disasters are responsible for the increasing number of orphans in low- and middle-income countries (LMICs).[2] Millions of non-orphaned children are separated from their biological parents either permanently or semi-permanently and are in need of supportive living environments. This separation often occurs because biological parents are: (a) unable to provide food, shelter, and safety; (b) forced to leave their children to seek employment elsewhere; or (c) physically or mentally unable to care for their children.[2]

The majority of OSC live in sub-Saharan Africa and Southern and Southeastern Asia, with Southern and Southeastern Asia having the largest number of orphans (61 million) while estimates for sub-Saharan Africa indicate that over 52 million children have been orphaned.[1] In other words, the countries with the highest rates of OSC are also among the economically poorest and most under-resourced.[2] Additionally, research suggests there are numerous negative outcomes associated with being an OSC in an under-resourced society, including traumatic grief, compromised cognitive and emotional development, less access to education, and a greater probability of being exploited for child labor.[3–10] Poverty extends into all areas of children’s lives and prevents children from having the security and structures required to grow, thrive, and develop.[9,11–14] OSC are in need of living environments that protect and promote their well-being.

Given the high rates of OSC and the extensive evidence on the negative outcomes associated with being an OSC in an under-resourced society, local and international communities have responded to this challenge by putting in place various alternative care options, including residential care (e.g. residential care centers [RCCs], group homes, “orphanages), and community-based family care (e.g. extended family member’s homes, adoption, foster care) in support of the affected children and their households. For children without adequate parental care, the international consensus is that there is a public responsibility to ensure alternative care is provided.[15]

As previously described in detail[16,17], the extent to which alternative residential care settings negatively affect children’s physical, cognitive, and psychosocial well-being has become a central debate for international aid policy affecting low- and middle-income countries (LMICs) with large numbers of OSC. Several oft-cited studies of infant children who lived in socially and emotionally deprived institutions in Europe[18–24] have concluded that institutional care is damaging to the development of young children relative to community-based foster care. Indeed, some studies demonstrated powerful negative effects of deprivation on infant development, and positive development when the infants were moved to live with well-trained and paid foster parents.[18,24] These studies of infants in a very specific and negative caregiving environment have been generalized to a belief that all residential care settings across the world must have the same poor caregiving characteristics: high child-to-caregiver ratios, shift work, low compensation for caregivers, regimented and non-individualized care, and a lack of psychological investment in the children.[25]

Accordingly, there is widespread belief and a plausible logic to support the premise that adequate care for OSC can be most effectively provided in community-based “family environments”; that is, settings that appear on their face to be similar to biological families (primary kin), and that may be especially well suited to meet the psychological needs associated with child development. Currently, global development guidelines and policies[26–29] recommend family-based care settings be considered first and “institutional <residential> care should only be used as a last resort”.[27]

However, studies that were designed to compare residential care centers to community-based family environments, and which include data from a broader array of cultural and situational contexts find more nuanced results. In several of these studies, children in residential care centers were found to fare as well as or better than those in community-based settings.[30–36] In addition, Whetten et al.[37] found that children in residential care centers across five LMICs (Cambodia, India, Kenya, Tanzania, and Ethiopia) fared better than those in community settings on several outcomes, including physical health, behavioral and emotional health, intellectual functioning, and memory. They also reported great variability between individuals within care settings (larger than variability between sites or variability between care settings within a site), and after adjusting for sites, age, and gender, discovered that residential care vs. community-based care settings explained only 0.3–7% of the variability in child outcomes.[37]

Certainly, when searching for the best alternative care option for orphan or separated children, most would agree that opportunities within the extended family or in other community- based settings should be seriously considered. However, as previously discussed[38], there is an important distinction between examining care options for OSC based on a priority scale, and carefully evaluating all options equally to determine the best fit for a child and their current needs. In practice, there has been movement from residential care centers to community family-based care without careful consideration of whether community family-based care settings better meet the needs of all children. The wide variety of reasons for which children find themselves living outside their family environment and in alternative residential care, as well as the numerous models and structures of alternative residential care available, motivates questions about how quality of care is defined and what features of care relate to child well-being, especially in under-resourced parts of the world.

Consequently, it is reasonable to believe that in under-resourced societies in LMICs with high and increasing rates of OSC, the principal functions of families for children may be more focused on the most basic needs.[39] Meeting these needs help children move toward more complex needs and develop positive child outcomes such as psychosocial well-being. Thus, the development of positive child outcomes (such as psychosocial well-being) may not be heavily dependent on membership in a community-based setting like a Western-style nuclear family. It may instead be dependent primarily on the availability of certain components of quality of care rather than the living environment itself where OSC receive care.

This study was designed to: (1) examine if the psychosocial well-being of OSC in under-resourced societies in LMICs is more dependent on the availability of certain components of quality of care rather than the care setting itself (i.e. residential care-based or community family-based setting), and (2) identify the relative importance of certain components of quality of care that are associated with a child’s psychosocial well-being across different OSC care settings.

While a small body of research suggests there may be certain components of quality of care that are linked to positive child outcomes[25,40,41], the current body of applicable research is limited in both quantity and scope, with no research that explores the relationship between components of quality of care, such as food security, quality of shelter, quality of caregiving, and access to health care services and child psychosocial well-being by different OSC care settings. Currently, these gaps in knowledge diminish our ability to understand the specific needs and effectiveness of current OSC care settings and intervention programs.

## Materials and methods

### Study description

Positive Outcomes for Orphans (POFO) is an ongoing longitudinal study following a cohort of children who were age 6 to 12 at baseline, living in residential care or family-based care settings in six sites in five low- and middle-income countries: Battambang District, Cambodia; Nagaland and Hyderabad, India; Bungoma District, Kenya; Kilimanjaro Region, Tanzania; and Addis Ababa, Ethiopia. Children were enrolled between 2006 and 2008 and followed biannually. Using the 36-month follow-up data, a total of 2,013 (923 residential care-based and 1,090 community-based) OSC study participants from all six sites were available for analysis.

### Study sample

The POFO study employed a two-stage random sampling methodology to identify a representative sample of 1,357 OSC living in residential care settings and 1,480 OSC living in community-based settings. To sample children in community-based settings, geographic or administrative boundaries were used to define sampling areas (clusters) within each site, and 50 clusters in each site were randomly selected. From these clusters, up to five eligible children ages 6–12 years were randomly selected. Eligible children were orphans, defined as children with one or both parents deceased, and separated children, defined as children who had been separated from their parents with no expectation of return. To sample children from residential care settings, defined as having at least five children from at least two different biological families not related to the caregivers and not in a family home, up to 20 centers per site were randomly selected from lists of all residential care centers in the region. Residential care centers provided lists of all children aged 6 to 12, and were approached sequentially until 250 children were enrolled, with up to 20 children randomly selected from each center. The full sampling strategy and characteristics of the sample have been reported elsewhere.[16]

### Data collection

Research ethics approvals for data collected in this study were provided by the Duke University Health System IRB as well as the following ethics review committees in the participating countries: the National Ethics Committee for Health Research (NECHR) in Cambodia; the Sharan IRB in India; the Indian Council of Medical Research (ICMR); the Kenya Medical Research Institute (KEMRI); the Kilimanjaro Christian Medical College Research Ethics and Review Committee (CRERC) in Tanzania; the National Institute for Medical Research (NIMR) in Tanzania; the Save Lives Ethiopia IRB in Ethiopia; the Stand for Vulnerable Organization IRB in Ethiopia; and the National Health Research Ethics Review Committee (NERC) in Ethiopia. Written and verbal consent was obtained from all children’s primary caregivers and assent was obtained from each participating child. More details regarding the interviewer training and data collection methods can be found in Whetten et al.’s previous study.[16]

### Research measures

#### Assessment of components of quality of care: The Child Status Index (CSI)

The Child Status Index (CSI) was developed as an easy-to-use tool to assess children’s current needs, monitor improvements in specific dimensions of child well-being, and identify areas of concern that can be served by program interventions.[42] The development of the CSI tool began in Kenya and Tanzania and involved a community participatory process with key stakeholders to derive the different domains and factors. After implementing feedback from several other countries, the tool was successfully field tested in Kenya and Tanzania for inter-rater reliability and construct validity.[42]

The tool is based on several child-centered and broader environmental factors and was organized under six domains: Food and Nutrition; Shelter and Care; Protection; Health; Psychosocial; and Education and Skills Training. For each domain, there are two different factors that represent potential areas of concern, and which may be modifiable with additional resources. Each of the 12 factors was rated on four levels of well-being, where higher scores indicated better child and care setting status in that area (i.e. 1 = very high risk; 2 = moderately high risk; 3 = moderately low risk; 4 = no risk).

#### Components of quality of care (CSI factors)

Among the twelve different factors of the CSI, the following four factors were used for analysis based on their relevance to the “components of quality of care” construct, the expert recommendation of the CSI creator, as well as empirical evidence that suggests their potential effects on OSC care settings.

#### CSI factor 1: Food security

The goal of this factor was for the child to have sufficient and nutritious food at all times of the year to grow well and to have an active and healthy life. “Food Security” was defined as: “the ability of the household or institution to obtain and provide enough food for the child. This food should be obtained through socially acceptable ways, without resorting to emergency food supplies, scavenging, begging, stealing, or other coping strategies”.[42]

#### CSI factor 3: Shelter

The goal of this factor was for the child to have a stable shelter that is adequate, dry, and safe. “Shelter” describes “the physical place or structure of the home or institution where the child lives and the extent to which the structure provides security, comfort and protection from weather. Stability is defined in terms of living in the same place for at least the past six months”.[42]

#### CSI factor 4: Caregiving

The goal of this factor was for the child to have at least one adult (age 18 or over) who provides consistent care, attention, and support. Caregiving was “seen as good when there is an identified adult (parent or guardian) who provides the child with a stable, nurturing, and emotionally secure environment. The relationship between the child and the caregiver should provide physical and psychological security for the child. This factor captured how committed the caregiver was to the child and to his/her involvement with the child”.[42]

#### CSI factor 8: Health care services

The goal of this factor was for the child to have access to health care services, including preventive care and medical treatment when ill. Adequate “health care services” was defined as “a child’s access to basic health care services that were age-appropriate, including immunizations (for children under five), bed nets, health education (e.g., HIV prevention for youth), other preventive measures, and appropriate medical care and medicines when sick”.[42]

#### Child psychosocial well-being

The Strengths and Difficulties Questionnaire (SDQ) [43] self-report was administered to children aged 10 and older. This brief behavioral screening tool (applicable for children 3–16 years old) was used to assess behavioral and emotional difficulties.

The four difficulties subscales of the SDQ (emotional symptoms, conduct problems, hyperactivity/inattention; peer relationship) can be summed to create a total difficulties score. There were 5 items in each subscale, and each item was scored from 0–2.

The SDQ was selected because of the dimensions of behavior assessed, its brevity, and its frequent use in studies of children in international contexts.[44–46] Although the SDQ has no published data regarding its psychometric properties or standardization in the five countries of this study, its validity is supported by translation and use in over 80 languages and the attention with which translations were conducted with native language speakers in each of the study sites. The current study used the Total Difficulties scale (20 items; α = .78) from the child self-report version [43] as a measure of psychosocial difficulties, with higher scores signifying more behavioral and emotional difficulties (ranging from 0–40).

### Analysis

Using data from the 36-month follow-up of the POFO study, a series of hierarchical linear regression analyses were used to test the main effects of the components of quality of care on child psychosocial well-being, while controlling for demographic factors. Age, gender, and orphan status (either “single” or “double” orphan or “separated”) were entered as the independent variables in the first model (Step 1) to control for available demographic factors. In the second model (Step 2), each of the variables of interest (care setting and each of the components of quality of care) was added separately as an independent variable to the analysis. R^2^ was used to assess explained variance as a way of understanding important predictors of SDQ.

Next, each of the CSI variables measuring quality of care were dichotomized to “high” and “low” categories, where CSI values of 4 were labeled “high” and CSI values of 1–3 were labeled “low.” Mean SDQ Total Difficulties scores were computed for both “high” and “low” CSI values for each care setting type (residential care and community-based settings). This process was done with each of the four CSI factors.

## Results

Table 1 describes the demographic characteristics of the 2,013 orphan and separated children (923 residential care-based and 1,090 community-based) included in the 36-month follow-up of the POFO study (Table 1). The mean age of the sample was 9.19 years old and there was a higher proportion of males (n = 1120, 55.6%) than females (n = 893, 44.4%). Of the four categories of orphans, the greatest number of children were paternal orphans (n = 920, 45.7%), followed by double orphans (n = 549, 27.3%), separated or abandoned children with no dead parent (n = 288, 14.3%), and finally, maternal orphans (n = 256, 12.7%). Table 1 also displays the sample sizes and means for key demographic factors across OSC care settings.

**Table 1:** Demographic characteristics across OSC care settings.

Demographic Characteristic	Residential Care-Based		Community-Based		All Settings
	N	% or Mean (SD)	N	% or Mean (SD)	N (%) or Mean (SD)
**Age**	923	9.15 (1.64)	1090	9.23 (1.58)	9.19 (1.60)
**Gender**					
Male	523	56.7%	597	54.8%	1120 (55.6%)
Female	400	43.3%	493	45.2%	893 (44.4%)
**Orphan Status**					
Double Orphan	365	39.5%	184	16.9%	549 (27.3%)
Maternal Orphan	92	10.0%	164	15.0%	256 (12.7%)
Paternal Orphan	303	32.8%	617	56.6%	920 (45.7%)
Separated or Abandoned (with no dead parent)	163	17.7%	125	11.5%	288 (14.3%)

Table 2 shows the results of each regression model that tested the main effect of the components on quality of care on child psychosocial well-being, while controlling demographic factors (“β” reflects the beta coefficient on the variable of interest, “SE” is the Standard Error on the coefficient of interest, and “Change in R^2”^ reflects the change in explained variance when the main exposure of interest (e.g. food security) is added to the model with all the other control variables). Findings suggested that greater levels of components of quality of care, including food security, quality of shelter, quality of caregiving, and access to health care services, significantly predicted better psychosocial well-being after controlling for demographic factors. Compared to the 0% variance in psychosocial well-being explained by care setting in this model, food security explained 13.4%, quality of shelter explained 10%, quality of caregiving explained 8.9%, and access to health care services explained 7.3% of the variance in psychosocial well-being. When we controlled for orphan status, age, and gender, we again found that components of quality of care predicted SDQ Total Difficulties better than care setting.

**Table 2:** Regression analyses for components of quality of care predicting SDQ total difficulties.

Model	β	SE	R^2^	Change in R^2^	% Variance Explained
Base Model (demographic factors)	-	-	0.002	-	-
1 (Care Setting)	-0.030	0.238	0.002	0.000	0.00%
2 (Food Security)	-2.523	0.143	0.136	0.134	13.40%
3 (Quality of Shelter)	-2.340	0.157	0.102	0.100	10.00%
4 (Quality of Caregiving)	-2.252	0.162	0.091	0.089	8.90%
5 (Access to Healthcare Services)	-2.162	0.172	0.075	0.073	7.30%

**Fig 1:**
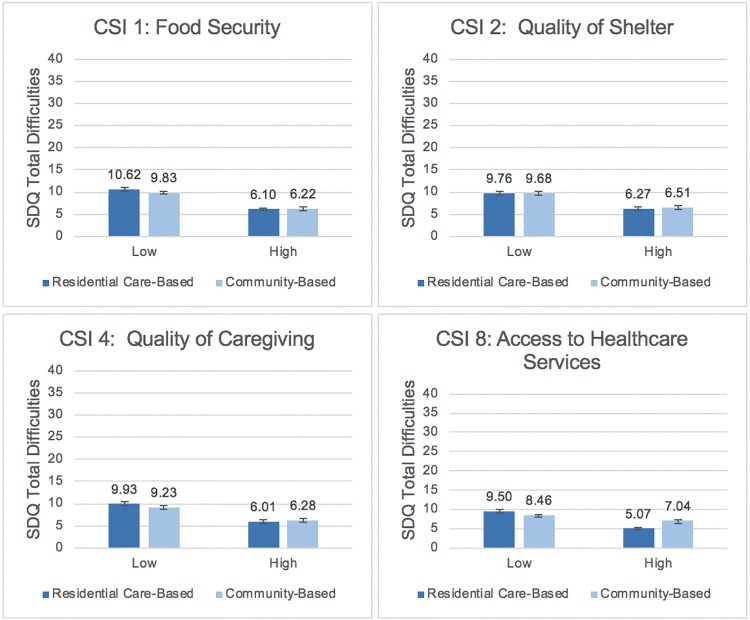
Relationships between components of quality of care and child psychosocial well-being by care setting.

After adjusting for demographic factors, mean SDQ Total Difficulties scores across “high” and “low” quality of care show differences between care settings to be minimal (Fig 1).

When levels of food security, quality of shelter, and quality of caregiving were “low” and “high”, there were no meaningful differences in SDQ total difficulties across care settings. When access to health care services was low, OSC in residential care-based settings had slightly higher SDQ total difficulties scores than OSC in community-based settings. However, when access to health care services was high, OSC in residential care-based settings had slightly lower SDQ total difficulties scores and therefore better psychosocial well-being than OSC in community-based settings.

Generally, findings suggest that when there are higher and lower levels of food security, quality of shelter, quality of caregiving, and access to health care services, the care setting was unrelated to psychosocial well-being. The mean SDQ total difficulties scores across “high” and “low” quality of care show differences between care settings to be minimal, and therefore are considered null results.

## Discussion

Findings from this study underscore the role of key components of quality care on child psychosocial well-being. Importantly, child psychosocial well-being did not vary by residential vs. family-based care within levels of high- and low-quality care. This study supported the hypothesis that, specifically in under-resourced societies in LMICs, psychosocial well-being for OSC may heavily depend on the quality of care provided within a setting rather than the care setting itself. Findings suggested that higher levels of all four components of quality of care (food security, quality of shelter, quality of caregiving, and access to health care services) significantly predicted more positive psychosocial well-being. Compared to the 0% variance explained by care setting in this current model, food security explained 13.4% of the variance in psychosocial well-being, while quality of shelter explained 10%, quality of caregiving explained 8.9%, and access to health care services explained 7.3%. Moreover, mean SDQ total difficulties scores across “high” and “low” levels of quality of care showed differences between care settings to be minimal, and are therefore considered null results.

It is reasonable to hypothesize that in LMICs with high and increasing rates of OSC, the principal functions of families for children may be more focused on their most basic and essential needs. Meeting these basic, survival needs help children move toward more complex needs and develop positive child outcomes such as better psychosocial well-being. Moreover, the delivery of such basic needs may not be heavily dependent on membership in a community-based setting like a Western-style nuclear family. It may instead be dependent primarily on the availability of certain components of quality of care rather than the structure or nature of the living environment where OSC receive care.

Thus, these findings cast doubt on conclusions from past studies indicating that residential care (institution-based) settings are systematically associated with poor child outcomes such as psychosocial well-being.[18–24] Instead, this study supports studies from a broad array of cultural and situational contexts suggesting that children in residential care centers may fare as well as or better than those in community-based settings.[30–36] Moreover, findings from this study suggest that in this study’s population across five LMICs, the psychosocial well-being of OSC in residential care settings (as measured by the SDQ) is no different from that of their community-based counterparts, and it is the availability of certain components of quality of care within the settings that make a difference. These findings should not be taken to mean that residential care settings are the better care setting for OSC, but rather that community-based care settings may perhaps not be all that different when it comes to predicting child psychosocial well-being. Given this, it is crucial that stakeholders create policies and practices that effectively support the improvement of quality of care across all OSC care settings.

This study has many important strengths, including the inclusion of six culturally diverse sites from LMICs, the rigorous sampling methodology that yielded statistically representative samples of residential care- and community-based OSC from each site, the longitudinal study design, and the high retention rate.[16] Several limitations should also be noted. Although the study included diverse LMICs, there is no representation from South America or Eastern Europe, where much of the earlier research on residential care originated. Recognizing that contexts are not necessarily interchangeable, continued study should focus on inclusion of other cultural contexts not represented in this study. Another limitation of this analysis involves the range of variables examined. Research suggests that there are other possible factors and characteristics that are significantly associated with OSC psychosocial well-being that were not included in this analysis, such as prevalence and incidence of trauma[47], school attendance[48,49], experience of HIV/AIDS stigma[50], socioeconomic status[51], caregiver health[52], or socio-cultural settings. Accordingly, it is possible that components of quality of care may act in tandem with other factors which influence psychosocial well-being, and further research is needed to tease out these relationships.

Although several limitations were identified, this analysis was innovative in its design. No previous studies have used a study sample of OSC across both residential- and community-based care settings to examine the extent to which certain components of quality of care predict child psychosocial well-being and how that varies across care setting. Findings suggest the potential of tools, such as the Child Status Index (CSI), to monitor and evaluate the quality of care within all care settings. Additionally, these findings offer a better understanding of where to intervene to improve orphan psychosocial well-being and suggest a focus on certain components of quality of care, such as food security, quality of shelter, quality of caregiving, and access to health care services. However, to truly understand how to improve these components of quality of care, more research is needed to identify and measure aspects of care that are specifically associated with high quality care and good child outcomes. Research to determine what high quality care and good child outcomes look like for specific demographics and socioeconomic and cultural contexts could elucidate intervention points that stakeholders need to effectively support the well-being of orphaned and separated children. Such research is essential as millions of children continue to live without parental care across both residential- and community-based care settings in under-resourced societies.
